# Validation of Plateletworks ADP for the ProCyte Dx analyzer

**DOI:** 10.1111/jvim.16670

**Published:** 2023-03-01

**Authors:** Matthew R. Kornya, Anthony C. G. Abrams‐Ogg, Shauna L. Blois, R. Darren Wood

**Affiliations:** ^1^ Department of Clinical Studies Ontario Veterinary College, University of Guelph Guelph Ontario Canada; ^2^ Department of Pathobiology Ontario Veterinary College, University of Guelph Guelph Ontario Canada

**Keywords:** aggregometry, clopidogrel, platelet aggregation

## Abstract

**Background:**

Platelet function testing in cats allows determination of clopidogrel effect. Plateletworks assesses aggregation based on decreasing platelet counts on hematology analyzers in response to agonists. It has not been validated for the IDEXX ProCyte Dx analyzer. Ideal time to perform analysis and the utility of other platelet parameters have not been fully assessed.

**Objectives:**

To validate Plateletworks ADP on the ProCyte Dx, to investigate the utility of various platelet parameters using Plateletworks ADP, and determine the ideal time to perform analysis.

**Animals:**

Twenty healthy cats recruited from the general population used for transference of reference intervals to a new analyzer, and 10 cats receiving clopidogrel to determine clopidogrel effect.

**Methods:**

Plateletworks ADP using the ProCyte Dx and ADVIA 2120i analyzer was run simultaneously in both healthy cats and cats receiving clopidogrel, and CBC results at different timepoints were compared between analyzers.

**Results:**

Aggregation was significantly different (*P* < .001) between analyzers. Cohen's kappa showed almost perfect agreement for determination of clopidogrel effect, and the area under the curve of the receiver operating characteristic was 1.0. Lower limits of the aggregation reference interval in healthy cats were 28.8% on the ProCyte Dx and 12.5% on the ADVIA 2120i. Coefficients of variation for platelet parameters were not different between analyzers. No significant changes in mean platelet volume, plateletcrit, large platelets, and mean platelet component were identified. No significant change in aggregation was observed within the first hour after phlebotomy.

**Conclusions and Clinical Importance:**

Our study validated the Plateletworks ADP system on the ProCyte Dx analyzer. Samples may be analyzed up to 1 h after collection.

**Abbreviations**
ADP: adenosine diphosphate
AUC: area under the curve
CAM: clopidogrel active metabolite
FeLV: feline leukemia virus
FIV: feline immunodeficiency virus
MPC: mean platelet component
PCT: plateletcrit
PLT: platelet count
ROC: receiver operating characteristic

## 1 INTRODUCTION

Thromboembolism is a life‐threatening consequence of hypertrophic cardiomyopathy in cats.^1^, ^2^, ^3^ The risk of thromboembolism may be decreased by the use of antiplatelet agents.^3^, ^4^ The antiplatelet drug most commonly used in cats is clopidogrel,^3^, ^5^ which permanently binds to the platelet membrane ADP receptor P2Y_12_ to prevent platelet degranulation, activation, and adhesion.^6^ However, in cats, dogs, and humans, clopidogrel resistance may be seen.^6^, ^7^, ^8^, ^9^ In humans, the rate of resistance has been reported as 8% to 40%,^10^, ^11^ and in cats from 15%^12^ to 50%.^13^ Resistance may be associated with multiple factors, including genetic variation in the CYP2C enzyme,^10^, ^14^ which converts clopidogrel to its active metabolite,^11^ effects of sex,^15^ and variations in the P2Y_12_ receptor.^7^, ^8^

Determining which cats are resistant to clopidogrel allows dose adjustment and initiation of alternative medications. Testing platelet function to identify nonresponders is crucial because the first clinical sign of platelet hyperreactivity is usually thromboembolism,^16^ which has a reported fatality rate in cats of 60% to 100%.^17^, ^18^, ^19^, ^20^ When thrombosis occurs in a patient receiving clopidogrel, platelet function testing is important to determine if a dose adjustment, drug class change, or dual antiplatelet treatment is indicated.

Routine coagulation tests such as prothrombin time, partial thromboplastin time, or thromboelastography cannot assess the effect of clopidogrel.^21^ Buccal mucosal bleeding time has been used as a point‐of‐care test of platelet function, but it may be difficult to perform in cats, is subject to equipment and operator variability, and has low repeatability.^22^, ^23^ Light transmission platelet aggregometry is the reference method to assess platelet function but is impractical for clinical use. As such, laboratory methods have been developed to more reliably, repeatably, and quantitatively assess platelet function. Most of these tests must be performed at point‐of‐care using equipment that is not economically feasible to maintain in veterinary clinics.

Plateletworks (Helena Laboratories, Beaumont, Texas) is based on the comparison of platelet counts in an EDTA‐anticoagulated sample to a sample treated with a platelet agonist (adenosine diphosphate, arachidonic acid, or collagen). Adenosine diphosphate is recommended to assess the effect of clopidogrel.^21^ Platelets counts determined by a hematology analyzer are compared between these samples. Lower percent aggregations are associated with effective clopidogrel treatment, whereas normal cats and those with clopidogrel resistance have higher percent aggregation.^24^

Plateletworks is currently validated in cats for a commercial reference laboratory optical analyzer (ADVIA 2120i; Siemens Healthcare, Toronto, Canada), as well as a point‐of‐care impedance analyzer (Abaxis HM‐5).^25^ The ProCyte Dx (IDEXX Laboratories, Markham, Canada) is among the more commonly used point‐of‐care analyzers and is an optical‐based technology.^26^ Plateletworks has not been validated for use on this analyzer.

Both the ADVIA 2120i and the ProCyte Dx report additional platelet parameters, such as mean platelet volume (MPV), plateletcrit (PCT), and (on the ADVIA only) mean platelet component (MPC). These have utility in the determination of platelet function.

Our primary objective was to validate the Plateletworks ADP assay for the ProCyte Dx hematology analyzer. Secondary objectives included determination of the time to peak aggregation and investigation of parameters other than percent platelet aggregation.

## 2 MATERIALS AND METHODS

### 2.1 Study design

Our study was designed as a prospective study to demonstrate equivalence of testing methodologies. This approach demonstrates the equivalence of a variation in a previously validated method of analysis without the need for de novo validation. Healthy cats were recruited from the students and staff of the Ontario Veterinary College Veterinary Teaching Hospital. Cats receiving clopidogrel were recruited from the clinical caseload of the hospital. Research was approved by the university Animal Care Committee (Animal Utilization Protocols 4252, 4356, 3587).

### 2.2 Case selection

Cats in the healthy group were confirmed to be healthy based on medical history, physical examination, and routine laboratory testing (CBC, serum biochemistry profile, and FeLV/FIV testing), and were excluded if they were receiving any medications known to affect coagulation or platelet function, including anticoagulants and antithrombotics, corticosteroids, antifibrinolytics, nonsteroidal anti‐inflammatory drugs, and thrombolytics. Clinical cats were receiving no medications affecting platelet function other than clopidogrel. Cats were excluded if their percent aggregation on the ADVIA 2120i was >25%, the previously determined lower end of the reference interval for healthy cats at our institution.^27^

### 2.3 Sampling methodology

Cats received gabapentin PO a median of 2.5 hours before phlebotomy. Doses ranged from 19.9 to 37.7 mg/kg (median, 26.5 mg/kg). The skin overlying both jugular veins was clipped, and a local anesthetic 4% lidocaine gel (Maxilene 4; Ferndale Laboratories Inc, Ferndale, Michigan) applied and covered with a bandage for a minimum of 30 minutes. Phlebotomy was performed immediately after removal of the bandage. The median time from gabapentin administration to local anesthetic application was 1 hour and 12 minutes, and the median time from local anesthesia to phlebotomy was 1 hour and 16 minutes. In cats that remained uncooperative and for which venipuncture without excessive platelet activation was considered to be difficult, butorphanol was administered IV via the medial saphenous vein at a dosage of 0.2 to 0.4 mg/kg followed by attempted venipuncture within 10 minutes.

Bandages covering the jugular veins were removed and the skin cleansed with isopropyl alcohol and allowed to dry. Cats were gently manually restrained and jugular venipuncture performed using a 22 gauge 1″ needle attached to a 6 mL syringe. Venipuncture and blood collection were subjectively graded by an observer using a scoring scheme.^28^ Briefly, initial venipuncture was either score 1: no redirection, immediate flow; score 2: 1 to 2 redirections; or 3: ≥3 redirections or unsuccessful aspiration. Blood flow was graded as 1: no stoppage of flow, smooth withdrawal; 2: a single brief flow interruption; or 3: multiple interruptions or redirection after flow started, and samples considered of insufficient quality were used for screening biochemistry and venipuncture repeated using the opposite jugular vein.

### 2.4 Platelet function analysis

Samples of adequate quality were immediately aliquoted into 1 mL Plateletworks ADP (20 μM ADP with 3.2% sodium citrate) and EDTA (7.5% K_3_ EDTA) tubes, that had been removed from refrigeration <30 minutes before phlebotomy. Lids were removed from the tubes (which do not contain a vacuum), the needle removed from the syringe, and the ADP tube filled by dispensing to the indicator line, followed by filling of the EDTA tube to the indicator line. Tubes were mixed by 15 full inversions. Transfer pipettes (Thermo Fisher Scientific) then were used to divide each sample into two 500 μL aliquots into no‐additive plastic tubes (BD Vacutainer), first filling the ADP sample tubes followed by the EDTA sample tubes.

Immediately after aliquoting, samples were manually transported to an on‐site reference laboratory for analysis using an ADVIA 2120i, and to an on‐site first opinion health care hospital for analysis using a ProCyte Dx. Quality control and priming of the ADVIA 2120i and initialization of the ProCyte Dx were performed before phlebotomy. Cell phone calls between operators of the 2 analyzers were used to precisely coordinate the timing of analyses. The ADP aliquot was analyzed first with a target time of approximately 10 minutes postcollection, followed by a second ADP analysis approximately 2 minutes later (based on the turn‐around time of the ProCyte Dx). The EDTA aliquots then were analyzed in duplicate approximately 2 minutes apart. The ADP aliquot then was analyzed again in duplicate with a target time of approximately 30 minutes postcollection. The first EDTA aliquot analysis by the ADVIA 2120i was performed with a full white blood cell (WBC) differential count (which uses 250 μL) to confirm health and allow comparison with the ProCyte Dx. The second EDTA analysis and all ADP analyses by the ADVIA 2120i were performed without a differential count to minimize required sample volume (175 μL) and allow for multiple analyses. All ADVIA 2120i samples were aspirated using the Manual Open tube sampler. All recorded time points were measured to the minute with values obtained from a cell phone.

### 2.5 Data analysis

Percent aggregation (%Agg) was calculated according to manufacturer recommendations using the equation:

$$\%\mathrm{Agg}=\left(\text{Platelet}\;{\text{Count}}_{\text{EDTA}}-{\text{Platelet Count}}_{\mathrm{ADP}}\right)/\text{Platelet}\;{\text{Count}}_{\text{EDTA}}\times 100\%.$$

Percent aggregation also was calculated replacing platelet count with PCT in the above formula to determine a PCT %Agg.

Blood films were prepared from both ADP aliquots and both EDTA aliquots in between first and duplicate analyses and stained with a modified Wright's stain using an automated slide stainer. Slides prepared from EDTA and 10‐minute ADP samples were reviewed by a single investigator (MK) blinded to patient and ADP/EDTA status. Samples were categorized as “no/minimal clumping” if there were no visible clumps or 1 to 2 small clumps and a subjectively normal platelet count; “mild clumping” if there were multiple small clumps or a single moderate‐sized clump and a subjectively normal platelet count; “moderate clumping” if there were many small clumps, multiple moderate‐sized clumps, or a small number of large clumps and a subjectively decreased platelet count; and “marked clumping” if there were several larger clumps and rare to no free platelets. Samples with more than mild clumping in EDTA were excluded from analysis.

To determine if there was an optimal time to peak aggregation, a subset of samples was analyzed at 45 minutes and 1 hour postcollection. Samples were only analyzed on the ProCyte Dx because of the smaller sample volume requirement (30 μL). Not all samples were analyzed at all time points, dependent on sample availability, access to the analyzer, and time constraints.

White blood cell count, hematocrit (HCT), hemoglobin concentration (HGB), mean cell volume (MCV), platelet count (PLT), PCT, MPV, MPC and number of large platelets (LPLT) were compared between EDTA and ADP samples. Two parameters (MPC and LPLT) were only available on the ADVIA 2120i.

### 2.6 Statistical analysis

For platelet count, %Agg, and PCT %Agg, data were assessed for normality using the Shapiro‐Wilk test. Because data were not normally distributed, median values were reported for all descriptive statistics. Because values were obtained in duplicate, median values for the 2 analyses were reported. Percent aggregation for both analyzers was compared and simple linear regression used to assess correlation. To determine if median values of percent aggregation were different, a 2‐tailed Wilcoxon signed‐rank test was used, with the *Z* statistic reported because 20 cats were present per group. The Mann‐Whitney *U*‐Test was used to compare the proportion of large platelets between groups. Reference value advisor^29^ was used to determine the reference interval of the healthy cat population. Because data were not normally distributed or symmetrical, the robust method was used after Box‐Cox transformation.

Receive operating characteristic (ROC) curves were constructed and areas under the curve (AUC) calculated. Optimal cut‐offs for determining clopidogrel effect were determined using the Youden method. Based on the optimal cut‐offs, all animals in the study were classified as either clopidogrel responsive or nonresponsive. The correlation between analyzers was calculated using Cohen's kappa statistic. Accuracy of analyzers in determining clopidogrel effect was compared using Fisher's exact test.

Correlation between coefficients of variation (CV) was assessed using Wilcoxon ranked‐sum testing. A Bland‐Altman plot was used to visually assess for agreement between the ADVIA 2120i and ProCyte Dx percent aggregation. The effect of time on platelet aggregation was assessed using a generalized mixed model.

SAS, R, R‐Studio, and Microsoft Excel were used for statistical analyses. Receiver operating characteristic (ROC) analysis was performed using an online tool (easyROC).^30^

## 3 RESULTS

### 3.1 Population

Twenty‐one healthy cats were recruited from students, faculty, and staff of the Ontario Veterinary College Veterinary Teaching Hospital, of which 1 was excluded because of clotting of the sample after collection, leaving 20 in the final analysis. Cats were 1 to 12 years of age (median, 4.5 years) and consisted of 14 domestic shorthairs, 2 domestic longhairs, 1 domestic medium‐hair, 1 Persian, 1 Russian Blue, and 1 Devon Rex. Ten cats were neutered males and 10 were spayed females. Weight ranged from 2.65 to 7.55 kg (median, 4.87 kg), body condition score ranged from 3/9 to 8/9 (median, 5/9), and all muscle condition scores were 3/3.

Ten cats also were recruited from the population of cats presented to the hospital receiving clopidogrel for clinical conditions. Cats ranged from 2 to 8 years of age (median, 3.5 years). Nine cats were male, and 1 was female. Cats were comprised of 6 domestic shorthairs and 4 domestic longhairs. Weight ranged from 3.2 to 6.8 kg (median, 4.4 kg). All cats received clopidogrel tablets PO at a dose of 18.75 mg/cat (range, 2.7‐5.8 mg/kg; median, 4.26 mg/kg).

### 3.2 Venipuncture and analysis

Of the healthy cat samples utilized, 18/21 venipunctures were considered good quality and 3/21 were moderate; 17/21 blood draws were considered good quality and 4/21 were moderate. One sample (from a spayed female domestic shorthair) had moderate platelet clumping noted in the EDTA sample based on low platelet counts with both the ProCyte Dx and ADVIA 2120i, as well as on blood smear review, with higher aggregation in EDTA than ADP, and as such was excluded from analysis. Two cats required butorphanol at a dosage of 0.2 mg/kg IV in addition to gabapentin.

Of the clopidogrel‐treated cats, 6/10 venipunctures were considered good quality and 4/10 were moderate; 8/10 blood draws were considered good quality and 2/10 were moderate. No cat required butorphanol.

### 3.3 Distribution

In healthy cats, platelet count, PCT, MPV, %Agg, PCT %Agg, MPC, and LPC were not normally distributed. The degree of platelet clumping on blood smear review was confirmed to be minimal in all EDTA aliquots, and moderate‐to‐marked in all ADP aliquots. In clopidogrel‐treated cats, platelet count, PCT, MPV, %Agg, and PCT %Agg, were normally distributed. The degree of platelet clumping on blood smear review was confirmed to be minimal in all aliquots.

The median, SD, and coefficients of variation for duplicate analyses for PLT, PCT, MPV, and %Agg for healthy cats on both analyzers are presented in Table 1. The median, SD, and coefficients of variation for duplicate analyses for PLT, PCT, MPV, and %Agg for clopidogrel‐treated cats on both analyzers are presented in Table 2.

**TABLE 1:** Descriptive statistics for selected platelet parameters in EDTA and Plateletworks ADP samples on both the ADVIA 2120i and ProCyte Dx for 20 healthy cats.

Value	Median	SD	Coefficient of variation
ADVIA 2120i EDTA PLT (×10^9^/L)	272	81.8	3.0%
ADVIA 2120i ADP PLT (×10^9^/L)	83.25	78.2	24.0%
ADVIA 2120i EDTA PCT (%)	0.36	0.14	2.0%
ADVIA 2120i ADP PCT (%)	0.12	0.10	7.0%
ADVIA 2120i EDTA MPV (fL)	13.88	1.8	1.0%
ADVIA 2120i ADP MPV (fL)	14.5	2.2	3.0%
ADVIA 2120i Aggregation (%)	70.5	27.8	9.0%
ProCyte Dx EDTA PLT (×10^9^/L)	290.25	86.2	3.0%
ProCyte Dx ADP PLT (×10^9^/L)	56.25	69.1	44.0%
ProCyte Dx EDTA PCT (%)	0.46	0.14	2.0%
ProCyte Dx ADP PCT (%)	0.09	0.10	23.0%
ProCyte Dx EDTA MPV (fL)	15.83	3.5	1.0%
ProCyte Dx ADP MPV (fL)	15.6	4.2	1.0%
ProCyte Dx Aggregation (%)	78.9	22.6	9.0%

**TABLE 2:** Descriptive statistics for selected platelet parameters in EDTA and Plateletworks ADP samples on both the ADVIA 2120 and ProCyte Dx for 10 cats receiving clopidogrel.

Value	Median	SD	Coefficient of variation
ADVIA 2120 EDTA PLT (×10^9^/L)	314.5	127.5	2%
ADVIA 2120 ADP PLT (×10^9^/L)	289.0	120.0	5%
ADVIA 2120 EDTA PCT (%)	0.43	0.14	4%
ADVIA 2120 ADP PCT (%)	0.43	0.14	5%
ADVIA 2120 EDTA MPV (fL)	16.0	4.3	1%
ADVIA 2120 ADP MPV (fL)	15.8	3.9	1%
ADVIA 2120 Aggregation (%)	6.6	5.9	23%
ProCyte Dx EDTA PLT (×10^9^/L)	328.3	154.5	2%
ProCyte Dx ADP PLT (×10^9^/L)	320.3	132.6	4%
ProCyte Dx EDTA PCT (%)	0.55	0.21	3%
ProCyte Dx ADP PCT (%)	0.53	0.19	4%
ProCyte Dx EDTA MPV (fL)	16.4	1.3	0%
ProCyte Dx ADP MPV (fL)	16.7	1.8	0%
ProCyte Dx Aggregation (%)	7.1	8.8	16%

### 3.4 Coefficients of variation

In healthy cats, coefficients of variation were similar between analyzers for EDTA samples for all platelet parameters investigated, with no significant differences in MPV (*P* = .08), PCT (*P* = .59), or PLT (*P* = .93). In clopidogrel‐treated cats, there also were no significant differences in EDTA CV values for MPV (*P* = .16), PCT (*P* = .68), or PLT (*P* = .43).

In healthy cats, coefficients of variation were very similar for ADP samples for all platelet parameters investigated, with no statistically significant differences in MPV (*P* = .06), PCT (*P* = .22), or PLT (*P* = .08). In clopidogrel‐treated cats, no significant differences were found in ADP CV values for PCT (*P* = .70), or PLT (*P* = .73), but the CV for MPV was significantly lower on the ProCyte Dx (*P* = .02).

### 3.5 Platelet parameters

In healthy cats, no significant differences were found in MPV between EDTA and ADP samples for either the ADVIA 2120i (*P* = .09) or the ProCyte Dx (*P* = .9). A significant difference was found in PCT between EDTA and ADP samples for both the ADVIA 2120i (*P* < .001) and the ProCyte Dx (*P* < .001), with the PCT significantly lower in ADP samples.

In clopidogrel‐treated cats no significant differences were found in MPV between EDTA and ADP samples for either the ADVIA 2120i (*P* = .6) or the ProCyte Dx (*P* = .47). A significant difference was found in PCT between EDTA and ADP samples for both the ADVIA 2120i (*P* < .001) and the ProCyte Dx (*P* < .001), with the PCT significantly lower in ADP samples.

Large platelet count only was reported on the ADVIA 2120i, and the absolute count was found to be significantly different between EDTA and ADP samples (*P* < .001). However, large platelets made up 7.1% of platelets in the EDTA samples, and 10.0% of platelets in the ADP samples, and these proportions were not significantly different (*P* = .21). The MPC also was reported by the ADVIA 2120i, but it was not different between EDTA and ADP samples in either healthy (*P* = .59) or clopidogrel‐treated (*P* = .44) cats.

In healthy cats, platelet count differed significantly between EDTA and ADP samples for both the ADVIA 2120i (*P* < .001) and the ProCyte Dx (*P* < .001). No significant difference was found in EDTA platelet count between the ADVIA 2120i and ProCyte Dx (*P* = .06), but the counts in ADP were different (*P* = .003), with the ProCyte Dx count being lower.

In clopidogrel‐treated cats, platelet count did not significantly differ between EDTA and ADP samples for either the ADVIA 2120i (*P* = .67) or the ProCyte Dx (*P* = .76). No significant difference was found between the ADVIA 2120i and ProCyte Dx in the EDTA (*P* = .67) or ADP (*P* = .73) platelet counts.

### 3.6 Aggregation results

**FIGURE 1:**
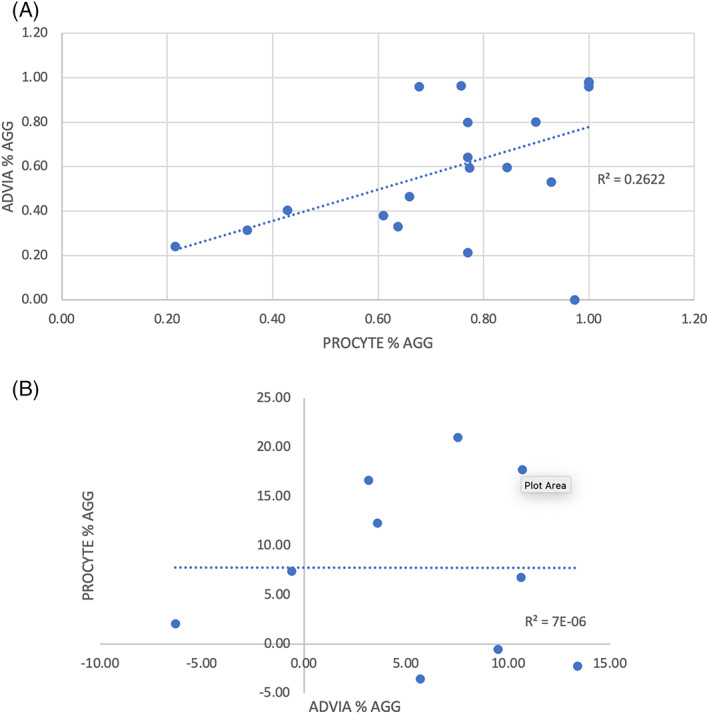
Correlation between Plateletworks ADP percent aggregation on the ProCyte Dx and ADVIA 2120i in (A) healthy cats and (B) clopidogrel‐treated cats.

In healthy cats, median %Agg was significantly different between the ADVIA 2120i and ProCyte Dx (*P* < .001) with the ProCyte Dx reporting higher values (ADVIA 2120i 16%‐100%; median, 27.8%; ProCyte Dx 34.6%‐100%; median, 22.6%). Simple linear regression for the association between aggregation on each analyzer is presented in Figure 1 (*r* ^2^ = 0.82).

In clopidogrel‐treated cats, median %Agg was not different between the ADVIA 2120i and ProCyte Dx (*P* = .73). Simple linear regression for the association between aggregation on each analyzer is presented in Figure 1 (*r* ^2^ < 0.001).

### 3.7 Agreement of analyzers

A Bland‐Altman plot for agreement between the 2 analyzers for %Agg was produced and evaluated (Supporting information S1). No outliers were identified and no fixed bias was noted.

Reference intervals were calculated using reference value advisor. On the ProCyte Dx, the reference interval for %Agg ranged from 28.8% (90% confidence interval [CI], 14.9%‐48.9%) to 125.9% (90% CI, 115.4%‐134.0%) with a median of 78.9%. On the ADVIA 2120i, the reference interval for %Agg ranged from 12.5% (90% CI, 5.9%‐26.4%) to 131.0% (90% CI, 115.8%‐140.8%) with a median of 70.5%. These reference intervals were different for the ADVIA 2120i and the ProCyte Dx.^25^

**FIGURE 2:**
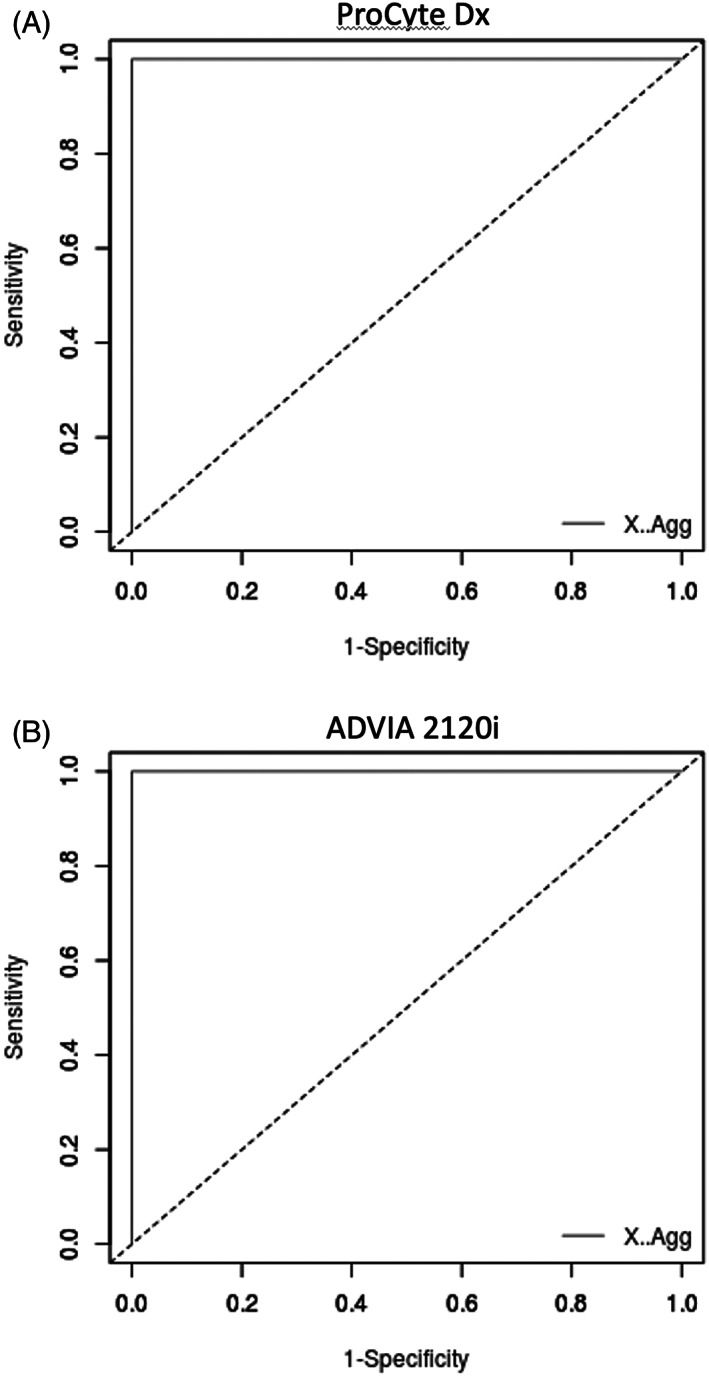
Receiver operating characteristic curves for both the (A) ProCyte Dx and (B) ADVIA 2120i for determination of clopidogrel effect with Plateletworks ADP. 191 × 186 mm (144 × 144 DPI).

Receiver operating characteristic curves were constructed for the determination of clopidogrel effect for both the ProCyte Dx and ADVIA 2120i (Figure 2). Both analyzers performed well, with overall identical performance. Ideal cut‐offs were determined using the Youden method. The ideal cut‐off for the ADVIA 2120i was slightly higher than the lower reference limit, but very similar (13.4% as a cut‐off vs 12.5% as the lower reference limit), and within the 90% confidence interval. The ideal cut‐off for the ProCyte Dx was slightly lower than the lower reference limit, but also very similar (21% as a cut‐off vs 28.8% as the lower reference limit), and within the 90% confidence interval. Area under the curve of the ROC, sensitivity and specificity were 1.0 for both analyzers.

The accuracy of the ProCyte Dx and ADVIA 2120i were compared to each other using both the lower reference limit and the ROC optimal cut‐off as threshold values for determining clopidogrel effect. Using the reference intervals, the ADVIA 2120i and ProCyte Dx each correctly classified cats according to study parameters in 29/30 cases (96.6%, *P* = 1), although a different cat was misclassified by each analyzer. Cohen's kappa for agreement between these tests was 0.85. Using the ROC cut‐off, the ADVIA 2120i correctly classified cats according to study parameters in all (30/30) cases, whereas the ProCyte Dx was correct in 29/30 (96.9%), which was not significantly different (*P* = 1). Cohen's kappa for agreement between these tests was 0.92.

**FIGURE 3:**
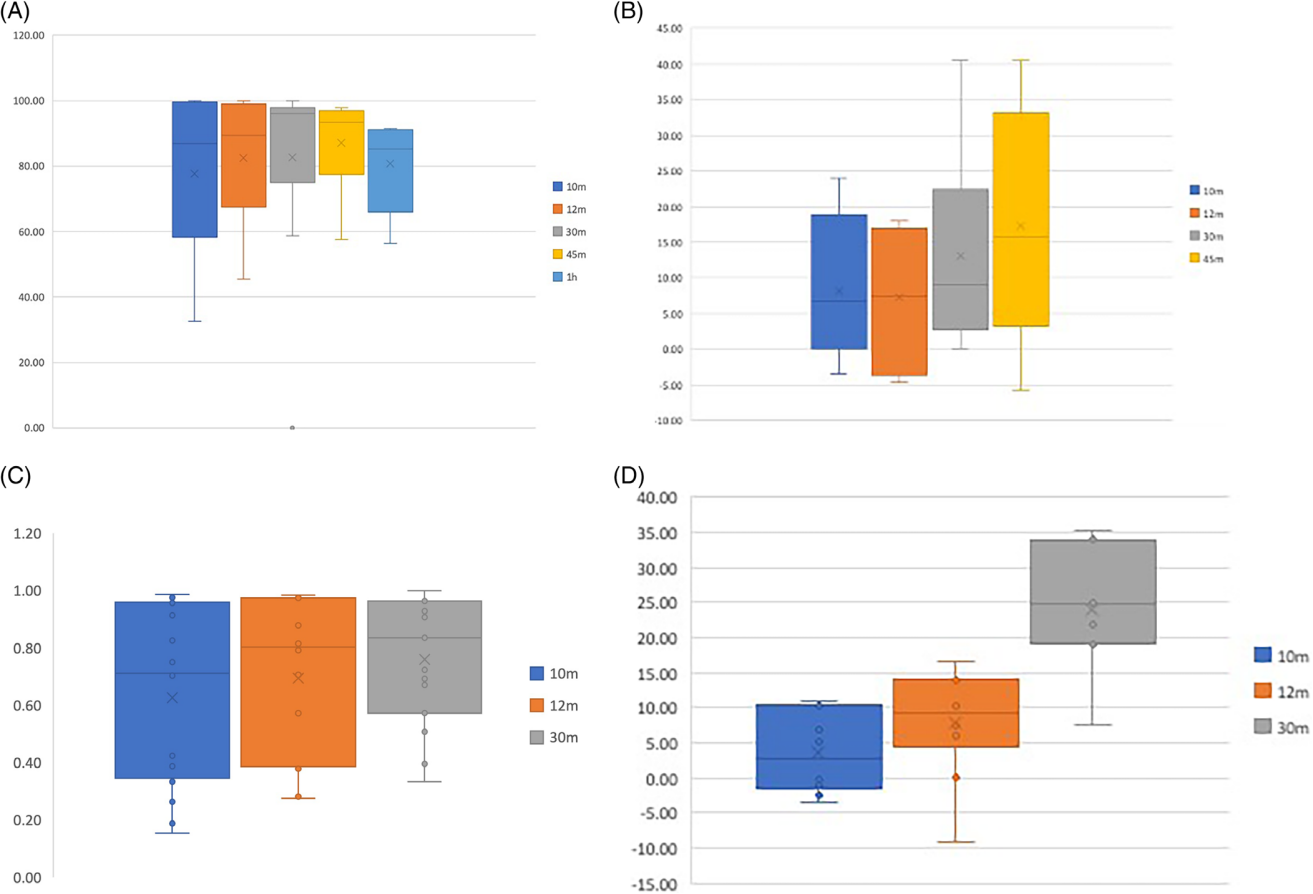
Box and Whisker plot showing percent aggregation of platelets over time with the ProCyte Dx in (A) healthy cats and in (B) clopidogrel‐treated cats; and with the ADVIA 2120i in (C) healthy cats and (D) clopidogrel‐treated cats. 268 × 191 mm (144 × 144 DPI).

For analysis of the effects of time on %Agg in healthy cats on the ProCyte Dx, 20 samples were available for analysis at 10, 12, and 30 minutes postcollection; 8 values at 45 minutes, and 7 values at 1 hour. Percent aggregation was compared over time (Figure 3A). The change over time was analyzed using a multiple effects model, and showed no significant difference (*P* = .12) at any timepoint.

### 3.8 Time to analysis

For analysis of the effects of time on %Agg in healthy cats on the ADVIA 2120i, 20 samples were available for analysis at 10 and 12 minutes and 15 samples at 30 minutes. Percent aggregation was compared over time (Figure 3B). The change over time was analyzed using a multiple effects model, and showed no significant difference (*P* = .17) at any timepoint.

For analysis of the effects of time on %Agg in clopidogrel‐treated cats on the ProCyte Dx, 10 samples were available for analysis at 10, 12, and 30 minutes postcollection; and 8 values at 45 minutes. Percent aggregation was compared over time (Figure 3C). The change over time was analyzed using a multiple effects model, and showed no significant difference (*P* = .54) at any timepoint.

For analysis of the effects of time on %Agg in clopidogrel‐treated cats on the ADVIA 2120i, 10 samples were available for analysis at 10 and 12 minutes and 7 samples at 30 minutes. Percent aggregation was compared over time (Figure 3D). The number of cats included was insufficient for meaningful statistical analysis.

## 4 DISCUSSION

The effect of clopidogrel on platelet function has been investigated using several methodologies in the past, but major logistical barriers appear to be present to the routine clinical use of these tests and they have not been widely adopted in clinical practice. The ability to use these tests more widely would be useful, because they help guide treatment and prevention for a devastating disease of cats.^31^ The results of our study will help to attain that goal.

When determining clopidogrel effect, both pharmacokinetic and pharmacodynamic assays are possible. Pharmacodynamic assays for clopidogrel effect such as those described here may have some advantages over pharmacokinetic assays, but they suffer from substantial limitations because of either intensive preanalytic requirements,^6^ substantial costs, or inability to transport samples for testing at a reference laboratory.^27^ Plateletworks offers an inexpensive test that requires no specialized equipment and theoretically may be performed in any veterinary hospital using a point‐of‐care analyzer. Major limitations to its widespread adoption include lack of validation on all common point‐of‐care analyzers, need for time‐sensitive testing, and uncertainty in interpretation of results.

Our results show that Plateletworks ADP may be analyzed using the ProCyte Dx with comparable results to the ADVIA 2120i, and as such may be performed in clinical practice. Although a significant difference between median values was noted between tests, using a ProCyte Dx specific reference interval resulted in almost perfect agreement between the tests. No bias or large outliers were observed when compared to the ADVIA 2120i.

Coefficients of variation were similar between analyzers for platelet count and %Agg. For MPV, the ProCyte Dx was comparable to the ADVIA 2120i. Because the CV for %Agg was the same in both analyzers and is the most relevant parameter, repeatability of testing was similar for both.

Previous Plateworks studies have evaluated platelet function testing up to 30 minutes postcollection, after which time spontaneous disaggregation was noted.^24^ The validity of testing at 30 minutes is consistent with results of our study, but no statistically significant change in %Agg was observed up to 1 hour postcollection. This finding may imply that a wider than previously recognized window of time is available for testing, increasing the practicality of this test in veterinary practice. Although a previous study in a smaller number of cats suggested a decrease in aggregation after 30 minutes,^24^ a similar effect was not observed in our study. This difference may be an artifact of small sample sizes, a result of different sample handling characteristics, or associated with phlebotomy technique. Further work is needed to confirm the feasibility of testing 30‐minutes postcollection.

Previous data has shown that Plateletworks ADP in cats has a lower reference value between 18% (with the HM5 analyzer) and 25% (with the ADVIA 2120i) aggregation.^25^, ^27^ Our lower reference limits of 12.5% to 28.8% are comparable to those previously reported. Interestingly, the lower reference limit for the ProCyte Dx more closely resembles the reference intervals previously determined for the ADVIA 2120i. Aggregation values >100% are not theoretically possible, and although the calculated reference interval extended above this value, in reality the upper limit should be 100%. Regardless, the upper reference limit is not clinically relevant, because platelet hyperreactivity is not detected by this test.

In addition to platelet count, several other platelet parameters are reported by the ProCyte Dx, and even more by the ADVIA 2120i. We showed that these values do not change significantly between EDTA and ADP samples, therefore limiting their use. Specifically, MPC held theoretical promise as a marker of platelet activation but did not do so in our analyses. Although the LPC was significantly different between EDTA and ADP samples, it correlated strongly with total platelet count and the percent of platelets that were large was not different between EDTA and ADP samples. As such, LPC provides no additional information compared to platelet count alone. Because LPC and MPC are not discriminating markers, the ADVIA 2120i does not provide additional information not otherwise available on the ProCyte Dx for Plateletworks testing.

A notable difference was observed in the lower limit of %Agg for the ProCyte Dx and ADVIA 2120i, but it does not seem to be relevant for determination of clopidogrel effect. The reason for this discordance is unclear, but may relate to the specific features of optical platelet counting in each analyzer. The tendency toward increased MPV in ADP samples compared to EDTA samples on the ADVIA 2120i but not the ProCyte Dx may suggest that the ADVIA 2120i detects smaller platelet clumps as platelets, whereas the ProCyte Dx does not. This difference may result in a higher platelet count with ADP samples (and therefore lower %Agg) on the ADVIA 2120i compared to the ProCyte Dx.

The main limitation of our study was the small sample size, but 20 individuals is considered sufficient for a study transferring an established reference interval to a new analyzer.^32^ A second limitation is that cats were sedated. Many drugs have been reported to affect platelet function, but it has become increasingly standard procedure to use sedation or anxiolytic drugs in cats before examination or phlebotomy.^33^ Both gabapentin and butorphanol have been administered to patients for platelet function studies in the past with no reported impact on function.^33^ It is also likely that cats in clinical practice will be given preexamination sedative or anxiolytic medication, and thus our data mimics a situation likely to be encountered by practicing veterinarians. In our study, the potential effects of sedative medications on platelet function were balanced against the prothrombotic effect of traumatic phlebotomy and difficult venipuncture, but they remain a potential limitation of the study. The number of cats enrolled in this study was not sufficient for generation of true reference intervals, however our study was not intended to determine de novo intervals for a new test, but rather to transfer a previously validated test to a new analyzer.

All cats receiving clopidogrel in our study were considered to have clopidogrel effect. However, the primary role of platelet function testing in cats is to determine if clopidogrel effect is present. Any cats with %Agg within the previously determined reference interval would have been excluded from our study, but none were detected in this group of 10 cats. This finding is not unexpected, because clopidogrel resistance rates of 15%^12^ may not be detected with only 10 cats. All cats receiving clopidogrel in our study also had platelet function determined by another analyzer (Platelet Function Analyzer‐200 [PFA]) as part of clopidogrel monitoring.^27^ One cat, with a %Agg of 7.5% on the ADVIA 2120i and 21% on the ProCyte Dx, was classified as resistant based on the PFA results. This cat was classified as having clopidogrel effect on all Plateletworks assays except the ProCyte Dx using the ROC generated cut‐off, which would have classified the cat as resistant. This discordant test result is not unexpected, because we have noted that some cats exhibit resistance on the PFA and not on Plateletworks, suggesting that the PFA may be a more sensitive but less specific test for clopidogrel resistance (unpublished data). More investigation in this area is needed.

Overall, we confirmed that Plateletworks ADP testing on the ProCyte Dx analyzer is a valid option for confirming normal platelet function in cats and has potential utility for determining the efficacy of clopidogrel treatment in cats. It further suggests that testing %Agg at 30 minutes may be superior to 10 minutes, and that values are not significantly changed for the first hour in some cats. Extension of the testing window to 1 hour postphlebotomy may not be valid for all cats based on previous studies, and further research in this area is needed. Finally, we confirmed that %Agg based on platelet number is the most relevant platelet parameter in determining platelet function in healthy cats using Plateletworks ADP, and that investigation of other parameters is unlikely to be of relevance.

## CONFLICT OF INTEREST DECLARATION

Authors declare no conflict of interest.

## OFF‐LABEL ANTIMICROBIAL DECLARATION

Authors declare no off‐label use of antimicrobials.

## INSTITUTIONAL ANIMAL CARE AND USE COMMITTEE (IACUC) OR OTHER APPROVAL DECLARATION

Approved by the University of Guelph Animal Use Committee, Animal Utilization Protocols 4252, 4356, 3587.

## HUMAN ETHICS APPROVAL DECLARATION

Authors declare human ethics approval was not needed for this study.

## Supporting information


**Figure S1:** Supporting information.
