# Supplementary material for: Validation of Plateletworks ADP for the ProCyte Dx analyzer
Source: J Vet Intern Med. 2023 Mar 1;37(2):518–27. doi: 10.1111/jvim.16670 (PMC10061176; doi:10.1111/jvim.16670)
Supplement: Supplementary file 1 — Figure S1: Supporting information. [file JVIM-37-518-s001.pdf]

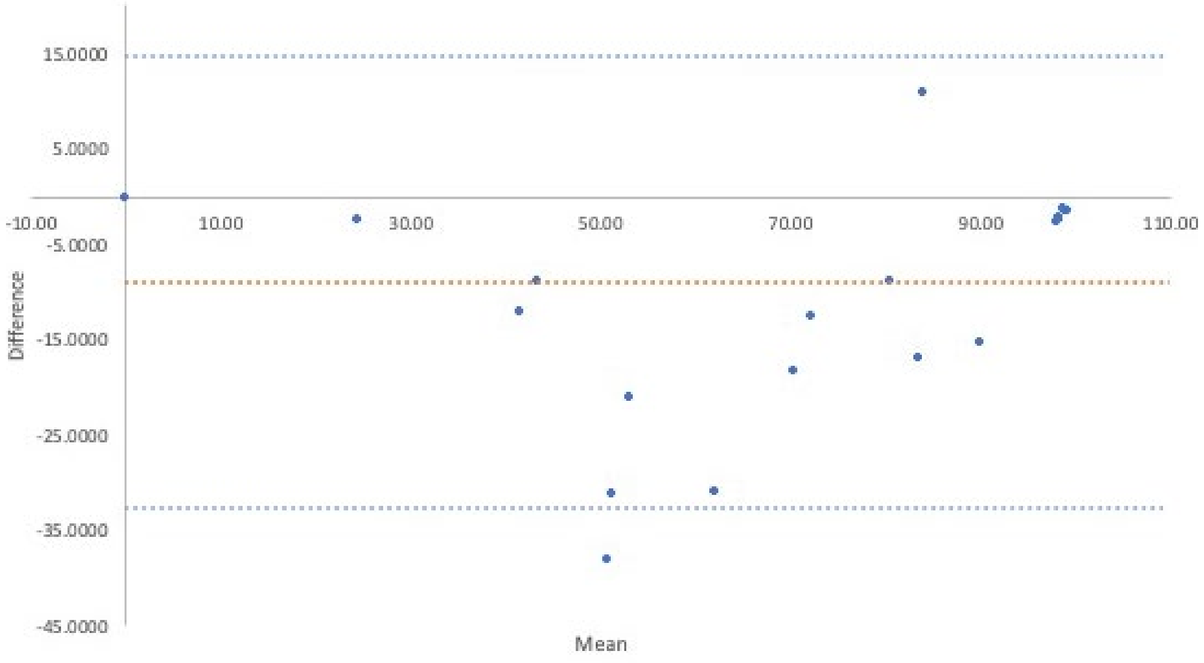

Bland-Altman plot comparing ProCyte Dx and ADVIA 2120i and Plateletworks ADP percent aggregation.

Horizontal lines show median (brown) and 1.96 standard deviations (blue).
